# Accuracy of a low-cost, portable, refractive error estimation device: Results of a diagnostic accuracy trial

**DOI:** 10.1371/journal.pone.0272451

**Published:** 2022-08-03

**Authors:** Sanil Joseph, Balagiri Sundar, Vinitha L. Rashme, Soundarya Venkatachalam, Joshua R. Ehrlich, Thulasiraj Ravilla

**Affiliations:** 1 Lions Aravind Institute of Community Ophthalmology, Madurai, India; 2 Centre for Eye Research Australia, Royal Victorian Eye and Ear Hospital, East Melbourne, Australia; 3 Department of Surgery (Ophthalmology), The University of Melbourne, Melbourne, Australia; 4 Department of Biostatistics Aravind Eye Hospital, Madurai, India; 5 Aravind Eye Hospital and Post Graduate Institute of Ophthalmology, Madurai, India; 6 Department of Ophthalmology and Visual Sciences, University of Michigan, Ann Arbor, MI, United States America; 7 Institute for Social Research, University of Michigan, Ann Arbor, MI, United States America; The University of Melbourne, AUSTRALIA

## Abstract

**Purpose:**

To assess the accuracy of refraction measurements by *ClickCheck*^*TM*^ compared with the standard practice of subjective refraction at a tertiary level eye hospital.

**Design:**

Diagnostic accuracy trial.

**Methods:**

All participants, recruited consecutively, underwent auto-refraction (AR) and subjective refraction (SR) followed by refraction measurement using *ClickCheck*^TM^ (CR) by a trained research assistant. Eyeglass prescriptions generated using *ClickCheck*^*TM*^ and the resulting visual acuity (VA) was compared to SR for accuracy. Inter-rater reliability and agreement were determined using Intra-class correlation and Bland Altman analysis respectively.

**Results:**

The 1,079 participants enrolled had a mean (SD) age of 39.02 (17.94) years and 56% were women. Overall, 45.3% of the participants had refractive error greater than ±0.5D. The mean (SD) spherical corrections were -0.66D (1.85) and -0.89D (2.20) in SR and CR respectively. There was high level of agreement between the spherical power measured using SR and CR (ICC: 0.940 (95% CI: 0.933 to 0.947). For the assessment of cylindrical correction, there was moderate level of agreement between SR and CR (ICC: 0.493 (0.100 to 0.715). There was moderate level of agreement between the VA measurements performed by using corrections from SR and CR (ICC: 0.577 (95% CI: 0.521–0.628). The subgroup analysis based on the age categories also showed high level of agreement for spherical corrections between the two approaches (ICC: 0.900). Bland Altman analysis showed good agreement for spherical corrections between SR and CR (Mean difference: 0.224D; 95% LoA: -1.647 D to 2.096 D) without evidence of measurement bias.

**Conclusions:**

There was a high level of agreement for spherical power measurement between CR and SR. However, improvements are needed in order to accurately assess the cylindrical power. Being a portable, low-cost and easy-to-use refraction device, *ClickCheck*^TM^ can be used for first level assessment of refractive errors, thereby enhancing the efficiency of refractive services, especially in low- and-middle-income countries.

## Introduction

Refractive error remains the most common cause of vision impairment, contributing to more than 53% of vision impairment [1, 2], and the second most common cause of blindness globally [3, 4]. Improved strategies to increase screening and correction for refractive errors could therefore have a substantial impact on reducing the global burden of vision impairment and associated socioeconomic losses.

Spectacle correction is the easiest and most economical treatment for refractive errors, but lack of easy access to refractive services and a limited numbers of trained professionals remain key challenges for uptake of spectacles in poorer regions of the world [5]. Most refractive error care relies on highly trained personnel such as ophthalmic technicians or optometrists to conduct refractions and provide prescriptions. These professionals are not available in many settings and where they are, their services add substantially to the cost of care [6].

Autorefractors, operated by eye care professionals or non-eye care health workers have been considered as an option to provide accurate refractions in various settings [7–12]. However, to date, autorefractors have been mostly used to assist optometrists or refractionists and the high cost and lack of portability of currently available devices have limited their use in resource constrained settings.

An easy to use, low-cost and portable device, the accuracy of which is non-inferior to the existing autorefractors may improve access to refractive error assessment, thereby better addressing visual impairment due to uncorrected refractive errors, especially in low-resource settings.

**Fig 1:**
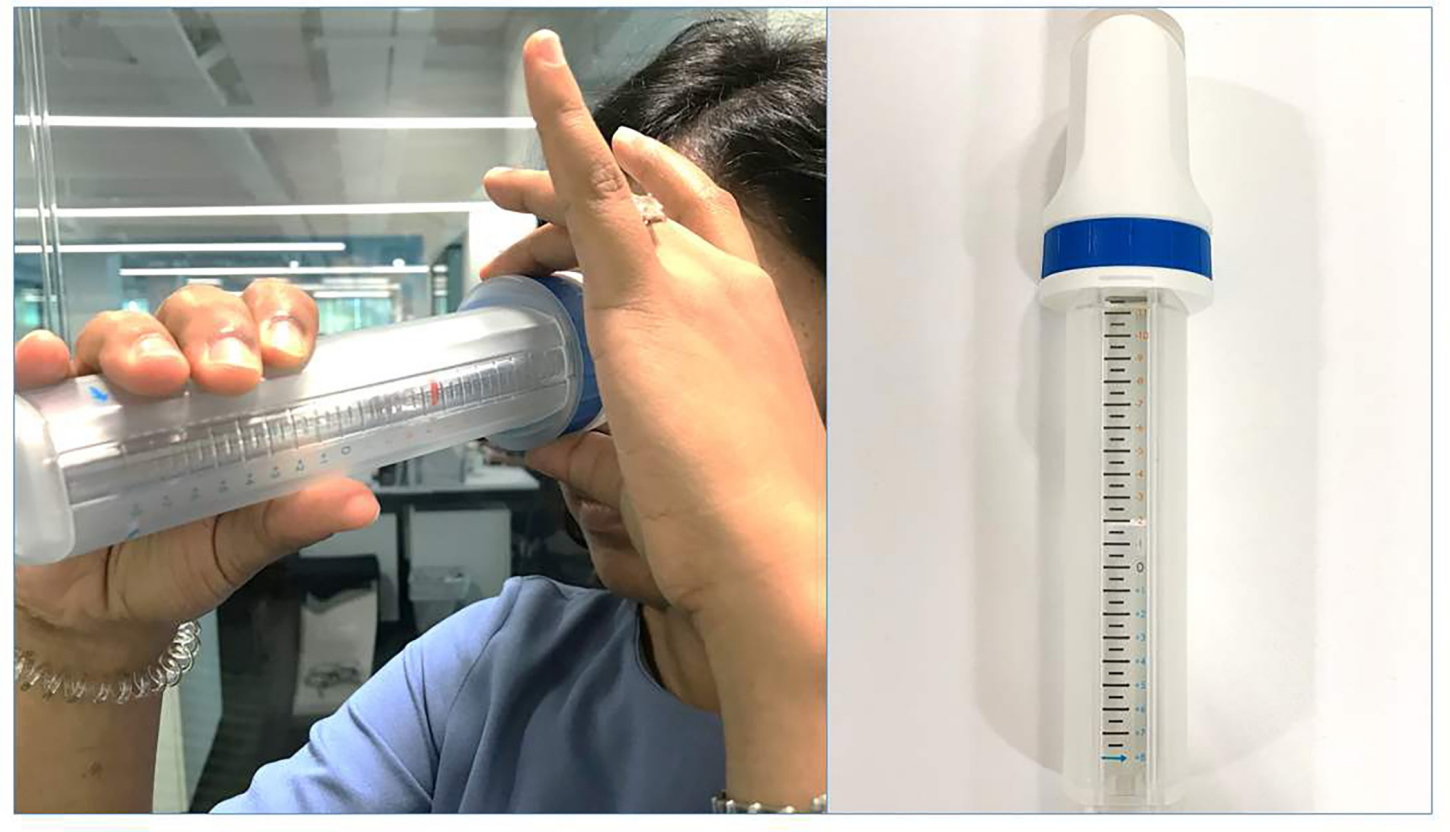
ClickCheck device and a patient being tested using the device.

*ClickCheck*^*TM*^ is a non-invasive, light weight device that can estimate refractive error of the user through adjusting the dial on a tube [13, 14]. Manufactured by Essilor International, the device is made of light weight plastic and can easily be sanitized just by wiping the tube and the eye piece [Fig 1]. This study aimed to assess the accuracy of refraction measurements using *ClickCheck*^*TM*^ compared with the gold standard practice of subjective refraction and an auto-refractor at a tertiary level eye hospital in India.

## Methods

We carried out a diagnostic accuracy study amongst patients visiting Aravind Eye Hospital (AEH), Madurai, India for refractive correction. Participants enrolled into the study underwent autorefraction and subjective refraction performed by an experienced refractionist, as per AEH’s standard protocol, followed by refraction measurement using *ClickCheck*^TM^ by a trained research assistant. Eyeglass prescriptions generated using *ClickCheck*^*TM*^ and the resulting visual acuity (VA) was compared with subjective refraction for accuracy.

The sample size was calculated as 887 participants assuming a sample agreement of 60%, power of 80% and 95% confidence interval for a two-sided test. Assuming a possible non-response rate of 15%, the target sample size was increased to 1,100 participants. The formula used for sample size calculation [15] is given below:

$$n=\frac{{\left(z_{\alpha }+z_{1-\beta }\right)}^{2}}{{\left\{(\rho_{1}-\rho_{0})P(1-P)\right\}}^{2}[\frac{1}{P^{2}+P(1-P)\rho_{0}}+\frac{2}{P(1-P)(1-\rho_{0})}+\frac{1}{{\left(1-P\right)}^{2}+P(1-P)\rho_{0}}]}$$

Where, P–Prevalence; ρ_0_–Population agreement; ρ_1_–Sample agreement; α–Level of significance; (1-β)—Power

The study was approved by the Institutional Ethical Committee at AEH and the study adhered to the tenants of the declaration of Helsinki throughout. The study protocol has been registered with Clinical Trial Registry of India (Registration No: **CTRI/2020/10/028564**).

### Inclusion and exclusion criteria

Participants aged between 7 to 70 years who attended the AEH General Ophthalmology clinic for refraction correction were recruited. Participants presenting with ocular injury or infections, media opacity with visual acuity <6/18 or nystagmus, refractive error beyond the measurement range of *ClickCheck*^TM^ (Spherical correction +8D to -11D and Cylindrical correction ≤ 4D), on medications that can influence refraction measurements, participants with physical, cognitive, or developmental disabilities were excluded.

### Study procedure

**Fig 2:**
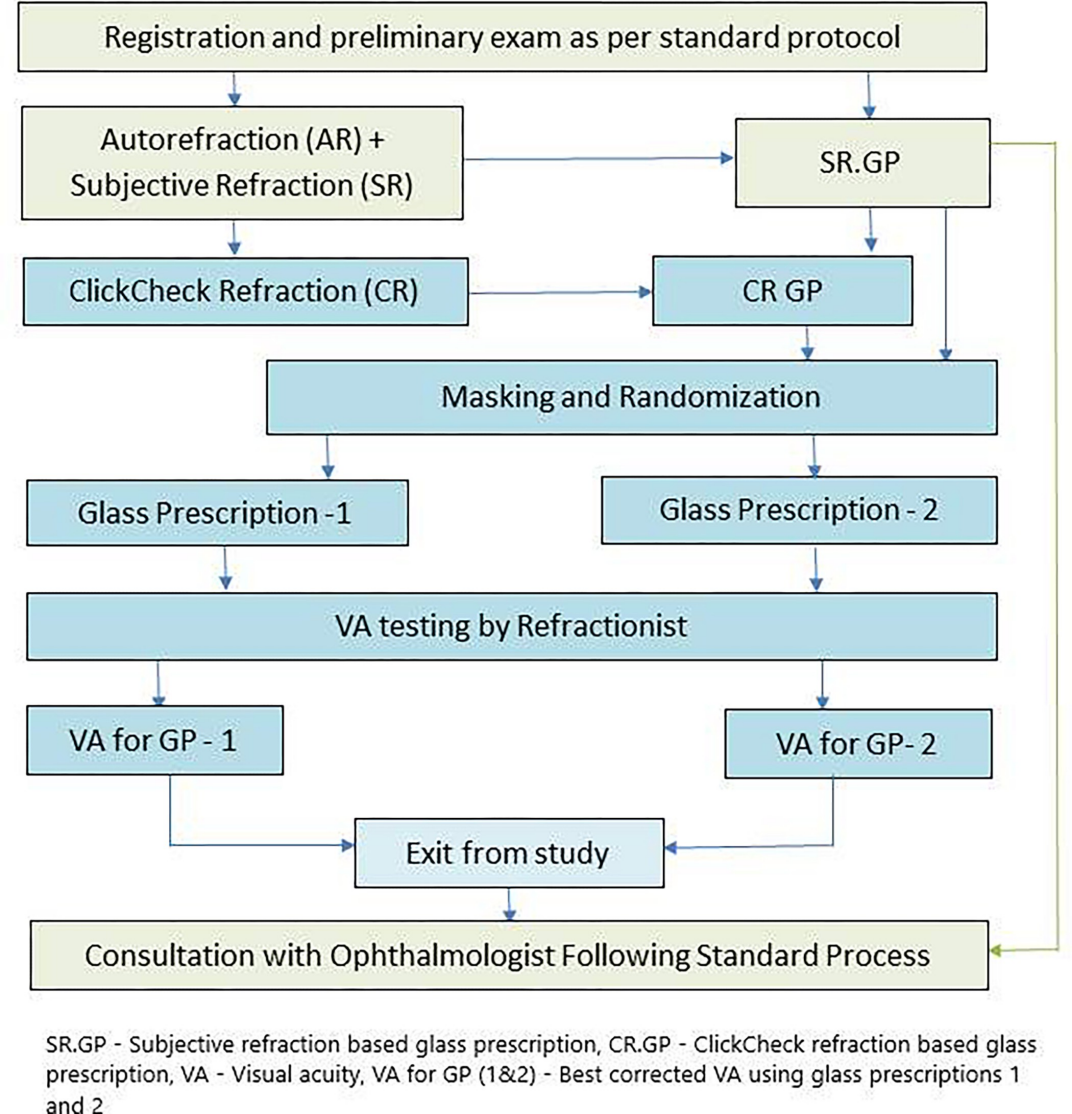
Study flowchart.

Fig 2 describes the process flow of the study. Participant registration, preliminary examinations, and subjective refraction (SR) were carried out following standard AEH procedures. Participants were recruited into the study after they went through the normal workflow for SR (Auto-refractor reading using Topcon AR 800, HQ: Tokyo, Japan followed by streak retinoscopy and subjective refraction). Informed consent was obtained along with the patient’s (or parent’s, in the case of paediatric participants) signature. Refraction estimation using *ClickCheck*^TM^ (CR) was carried out as per the procedure detailed below. Participants were given treatment advice based on the prescription from standard subjective refraction. Data analysis was done to compare accuracy of CR and SR.

### Estimation of refraction values using ClickCheck^TM^

Before starting the measurement, the research assistant briefed the participants about the device and the measurement process. Two research assistants, who were trained using a structured curriculum, performed the measurements. A standardised script was used to explain the procedure to the participants and the assessment was carried out first in the right eye followed by the left eye. For each eye, spherical power estimation was performed first followed by assessment for astigmatism and cylindrical power estimation, when indicated [16]. The time taken for the assessment was recorded using a stopwatch.

### Spherical power estimation

The ClickCheck device uses the principle of the Badal optometer [17]. The two important components of the Badal optometer include a convergent lens of focal length ‘f’ and an eye chart which can move along the optical axis of the convergent lens. Before taking the measurement for each eye, the eye chart on the device was set at the starting position (+8) by rotating the dial. The research assistant ensured that the occluder properly covered the fellow eye. After correctly placing the device on the eye, the participants were asked to look straight and focus at the centre of the vision chart. The vision chart contained axis lines that were 15° apart with a big letter ‘E’ (size 0.580mm) at the centre surrounded by four small letter ‘E’s (size 0.145mm) around it. Participants were then asked to rotate the dial clockwise until the fixation target (letter ‘E’s) in the middle of the chart became clear for the first time. When rotated the dial moves from +8D to -11D, in steps of 0.5D. The corresponding spherical value was recorded and this step was repeated three times and the average spherical value was calculated and recorded (A).

### Astigmatism assessment

To start the astigmatism assessment, the dial was kept at the average spherical value and +1 D was added by rotating the dial two steps anti clock-wise. Participants were asked to report whether they saw all the lines on the chart equally blurred, equally sharp, or if some of the lines were sharper than others. If the patient saw all the lines equally blurred or sharp, then the interpretation was that there was little (<1D) or no astigmatism. In that case, the measurement for that eye was completed and the patient was asked to use the device on the other eye. If the patient saw some lines sharper or more blurred than the others, then the interpretation was that there was astigmatism ≥1D. For participants with astigmatism ≥1D, the axis corresponding to the most blurred line was recorded as the axis of astigmatism (each line was separated by 15°) and then the cylindrical power estimation was performed.

### Cylinder power estimation

Participants were asked to continue rotating the dial clockwise until they saw all the lines equally clear and the corresponding value on the eye chart was recorded (B). Cylinder power was estimated by subtracting the new reading (B) from the average spherical power (A). The measurement for ‘B’ is repeated and subtracted from A three times. The average of the three measurements was recorded as the cylinder power. All the measurements were finalised by adjusting the dioptre power values to the nearest integer.

### Comparison of visual acuity (VA)

A refractionist, who was masked to the source of the refraction, tested VA for each patient using the glass prescriptions from CR and SR. The prescriptions were assigned a random serial number to ensure that the refractionist was completely masked to the source of refraction. After testing VA for each glasses prescription, the refractionist entered the values in the specified area in the database using login credentials.

### Statistical analysis

Intra-class correlation (ICC) along with 95% confidence Interval was used to validate the accuracy of refraction measurements from CR against those from SR and AR respectively. Bland Altman Plots were used to present graphically the level of agreement between the measurements approaches. We computed the 95% limits of agreement between the measurements using the approximation of the average difference ± (1.96 × SD) of the differences. Comparison of the mean spherical and cylindrical values across SR, CR and AR was conducted using analysis of variance (ANOVA). Bonferroni method was used to adjust for comparisons across these post hoc tests. Test-retest variability of ClickCheck device was assessed using repeatability coefficient (RC) and Intraclass correlation coefficient (ICC). RC was defined as 2.77 x s_w_, where s_w_ is the square root of within subject variance. The sensitivity, specificity, positive predictive value (PPV) and negative predictive value (NPV) were calculated comparing the spherical equivalent (SE) refractive error. The ability of CR in detecting refractive error (>0.5D) was evaluated by plotting a receiver operating characteristic (ROC) curve. An additional analysis was carried out to compare the spherical refractive error measurements from CR with SR and AR by age categories. All the analysis were performed using right eye data for each participant after establishing high level of correlation between the refractive measurements of the right and left eyes; ICC: 0.948 (95% CI: 0.941–0.954). We measured visual acuity using Snellen VA chart and the measurements were converted to LogMar values, using standard conversion chart, for assessing intra class correlation. All the analyses were performed using STATA version 14.0 and p <0.05 was considered as statistically significant.

## Results

The 1,079 eligible participants recruited into the study had a mean (SD) age of 39.0 (17.9) years. Approximately 56% of participants were female. Based on SR measurements, the spherical equivalent refractive error ranged from -12.5D to +5.5D and 477 (44.0%) had refractive error greater than ±0.5D [S1 Table in S1 File].

### Spherical correction

In total, 958 participants had spherical power measurements recorded in the right eye for all the three refractions (SR, AR and CR). The mean (SD) spherical correction in the right eye was -0.66D (1.85), -0.66 D (2.18) and -0.89 D (2.20) by SR, AR and CR, respectively and the difference in the means was found to be statistically significant (p = 0.026). The ICC value of 0.940 (95% CI: 0.933 to 0.947) and 0.965 (95% CI: 0.960 to 0.969) shows a high level of agreement for CR when compared with SR and AR, respectively [Table 1].

**Table 1:** Comparison of spherical, cylindrical correction and visual acuity using SR, AR and CR.

	**SR**	**AR**	**CR**	**P value**	Comparison between all the three refractions	**ICC (95% CI)**	**Mean difference (95% CI)**	**LoA**
**Spherical correction (n = 958)**						0.940 (0.933–0.947)^1^	0.224 (0.165 to 0.283)^1^	-1.647 to 2.096^1^
*Mean (SD)*	-0.66 (1.85)^a^	-0.66 (2.18)^a^	-0.89 (2.20)^a^					
*Min—Max*	-10.0 to 5.5	-11.5 to 6.25	-11.5 to 6.0	0.026†		0.940 (0.933–0.947)^1^	0.224 (0.165 to 0.283)^1^	-1.647 to 2.096^1^
**Cylindrical correction (n = 48)**						0.493 (0.100 to 0.715)^1^	-0.130 (-0.562 to 0.302)^1^	-3.105 to 2.845^1^
*Mean (SD)*	-1.34 (1.52)^b^	-2.52 (1.55)^b^	-1.21 (0.98)					
*Min—Max*	-4.5 to 2.5	-6.75 to 1.25	-4.00 to 0.00	<0.001†		0.306 (-0.233 to 0.610)^**2**^	-1.307 (-1.694 to -0.921)^2^	-3.968 to 1.353^2^
**Visual acuity (n = 958)**						0.577 (0.521–0.628)^1^	-0.125 (-0.135 to -0.114)^1^	-0.460 to 0.211^1^

^1^Comparison between SR and CR

^2^Comparison between AR and CR; SR-Subjective Refraction; AR-Autorefraction (Topcon); CR-ClickCheck Refraction; ICC–Intra-class correlation, LoA–Limit of agreement, CI–Confidence Interval

†ANOVA, NA- Not available

^a^No statistical significance was found in spherical corrections between SR and AR using Bonferroni corrections.

^b^ No statistical significance was found in cylindrical correction between SR and CR using Bonferroni corrections.

The repeatability coefficient for spherical values measured by ClickCheck was 0.68 D (95% CI: 0.62–0.74) which suggests that the absolute difference between the three measurements of CR on a subject would not differ by more than 0.68D on 95% of occasions. The intra-class correlation, ICC = 0.995 (95% CI: 0.989–0.997) denotes very high correlation of the three repeated spherical values measured using CR.

**Fig 3:**
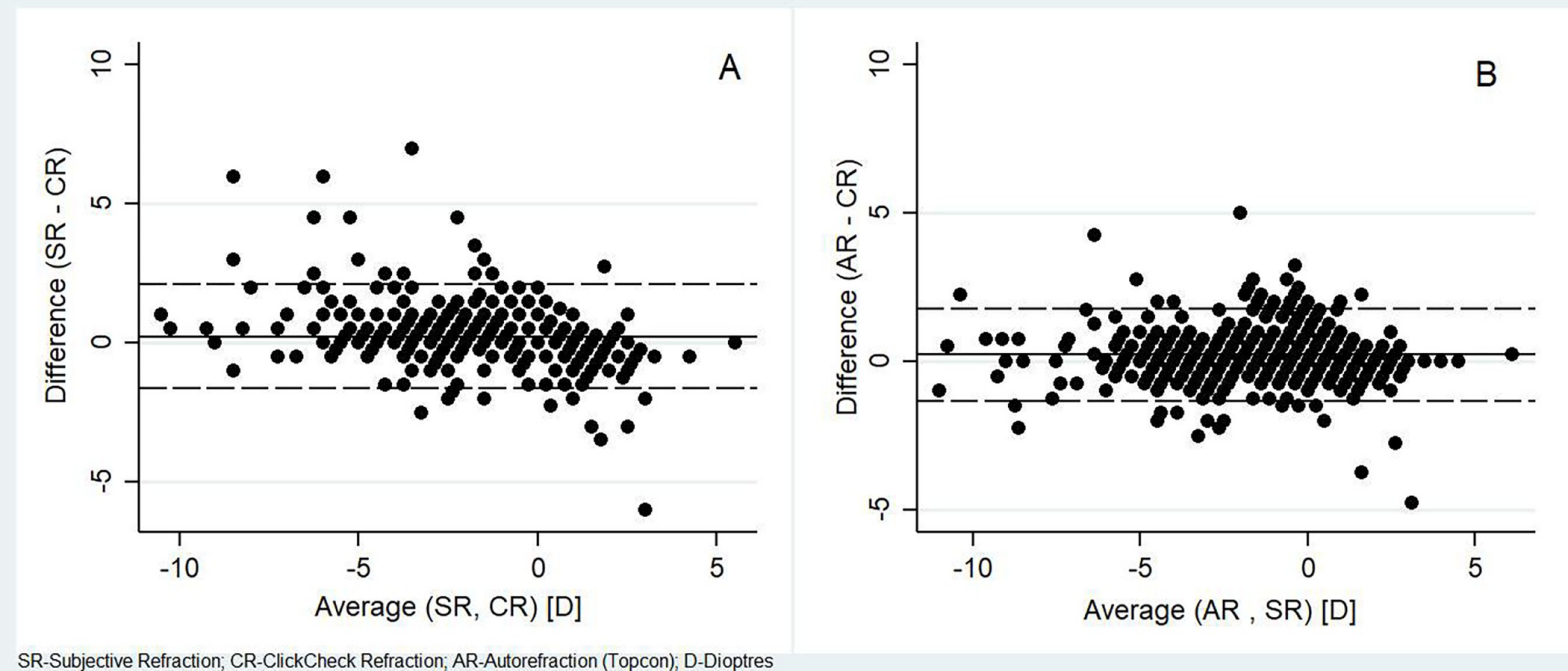
Bland Altman plot comparing the agreement of measurements by subjective refraction (SR), Conventional autorefraction (AR) and ClickCheck^TM^ refraction (CR): A) Subjective refraction vs ClickCheck refraction, B) Auto refraction vs ClickCheck refraction.

From Bland-Altman analysis, we observed a difference between SR and CR of 0.224 D (95% LOA; -1.647 to 2.096), with CR showing higher myopic spherical values compared to SR. We found a similar measurement difference between the AR and CR of 0.222 D with a narrower LOA (-1.327 to 1.770) [Fig 3]. Thus, there was no evidence of systematic measurement bias.

### Cylindrical correction

ClickCheck detected 48 eyes as having astigmatism >1D with the means (SD) for cylindrical correction according to SR, AR and CR: -1.34D (1.50), -2.52D (1.55) and -1.21D (0.98), respectively and the difference among the means was found to be statistically significant (p <0.001). The ICC value of 0.493 (95% CI: 0.100 to 0.715, p = 0.010) and 0.306 (95% CI: -0.233 to 0.610, p = 0.105) shows a moderate and low level of agreement for CR when compared with SR and AR respectively. We observed a difference between the SR and CR of -0.130D (95% LoA: -3.105 to 2.845), with CR reporting lower cylindrical corrections than SR [Table 1]. However, ClickCheck detected significantly lower number of eyes to have cylindrical correction (48 eyes) compared to SR that detected 513 eyes.

**Fig 4:**
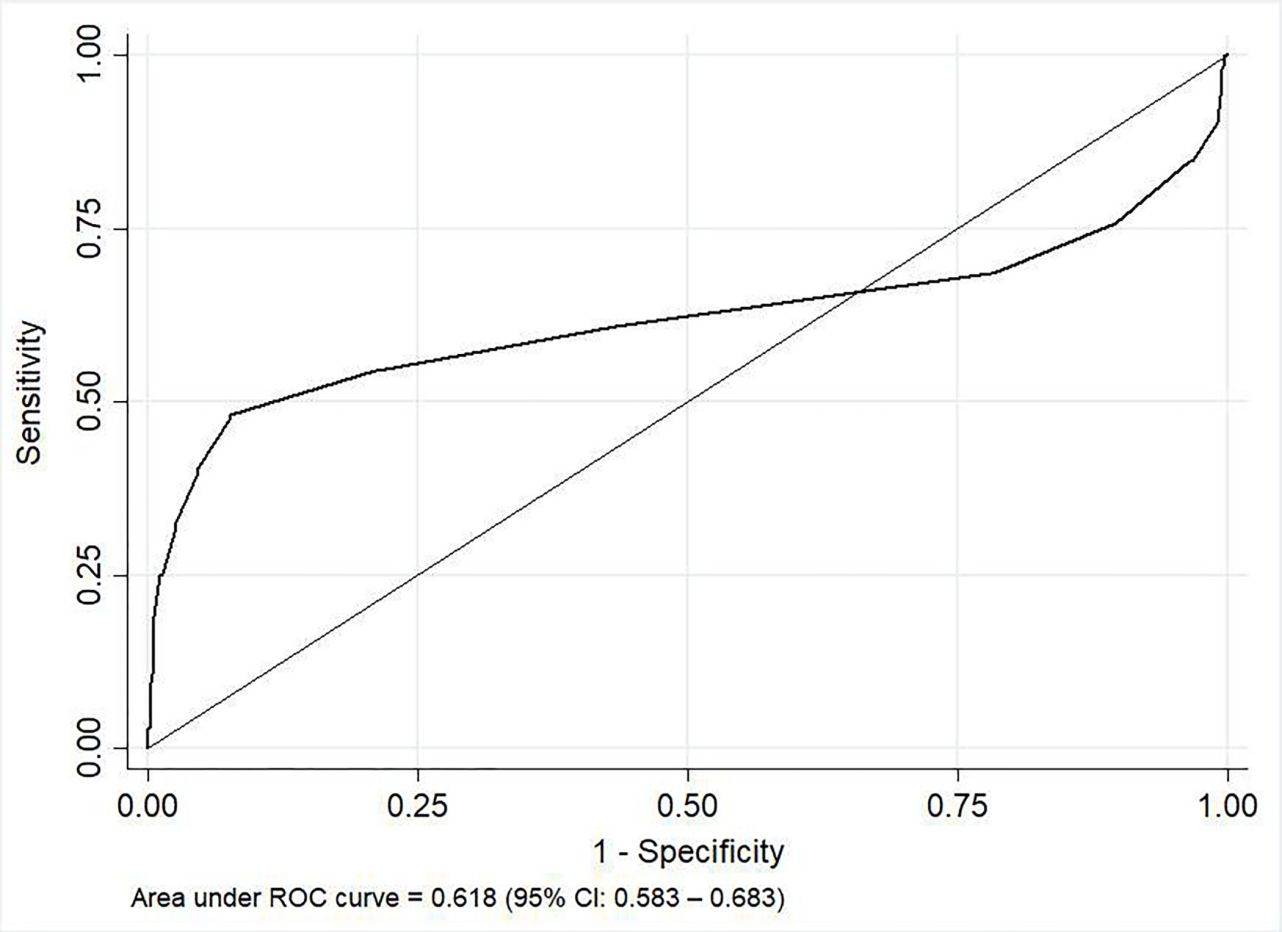
ROC showing the ability of CR in correctly differentiating eyes with refractive error (>0.5D) from eyes without refractive error.

Based on spherical equivalent (SE) refractive error, the sensitivity, specificity, PPV and NPV of CR against SR were 78.7%, 68.2, 82% and 63.5% respectively. The area under the ROC (AUROC) value of 0.618 (95% CI: 0.583–0.683) indicates a low ability of CR in correctly differentiating eyes with refractive error (> 0.5D) from eyes without refractive error [Fig 4].

### Visual acuity

The Mean (SD) Best corrected visual acuity (BCVA) based on SR and CR was 0.06 (0.14) and 0.18 (0.21) respectively [Table 1]. The ICC value of 0.577 (95% CI: 0.521–0.628, p < 0.001) shows a moderate level of agreement between the VA measurements with respect to SR and CR respectively.

### Subgroup analysis using age categories

**Fig 5:**
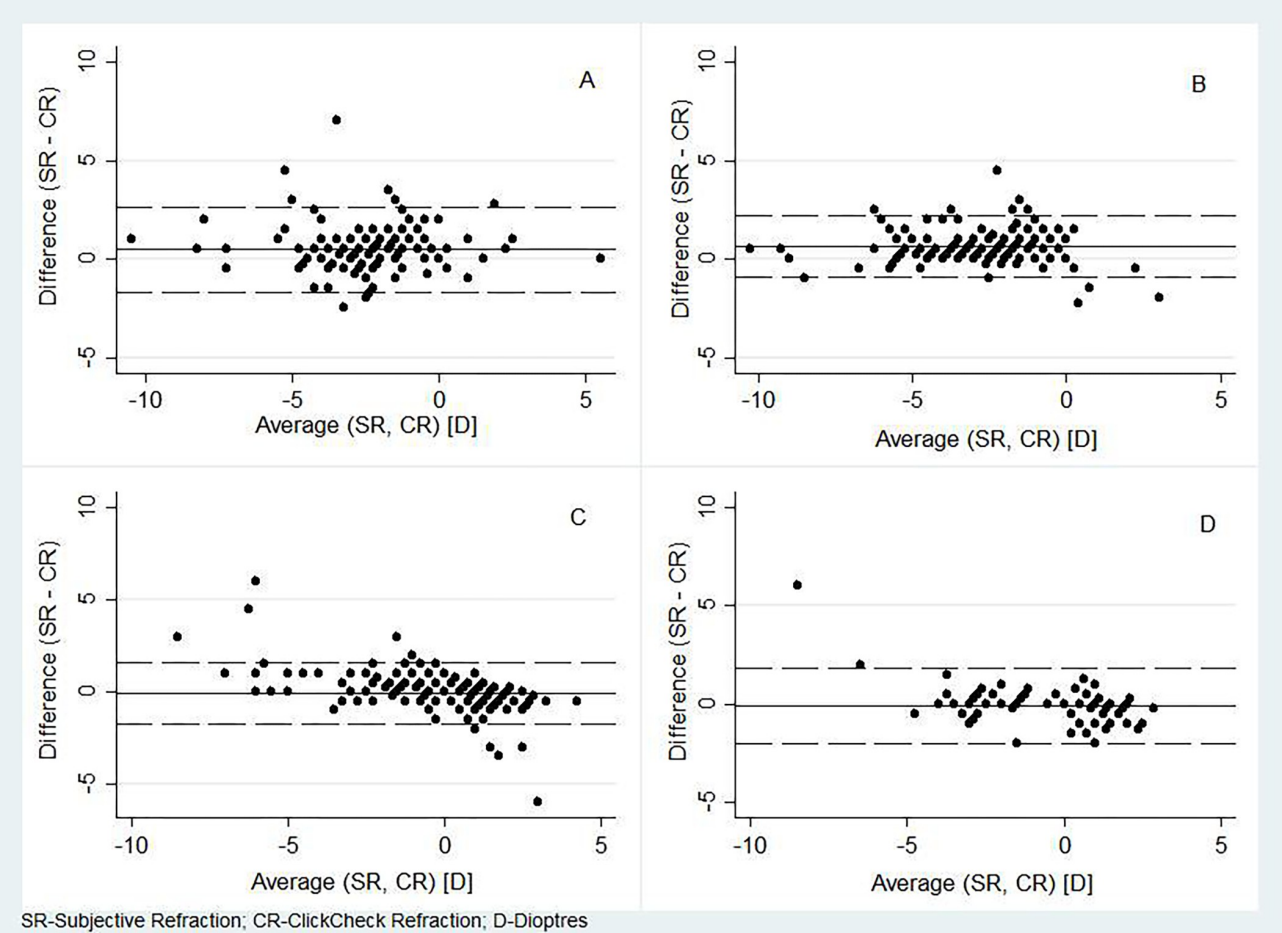
Bland Altman plot comparing the agreement of measurements by subjective refraction (SR) and ClickCheck^TM^ refraction (CR) across different age categories A) 7 to 15 years, B) 16 to 40 years, C) 41 to 59 years, D) 60 to 70 years.

**Fig 6:**
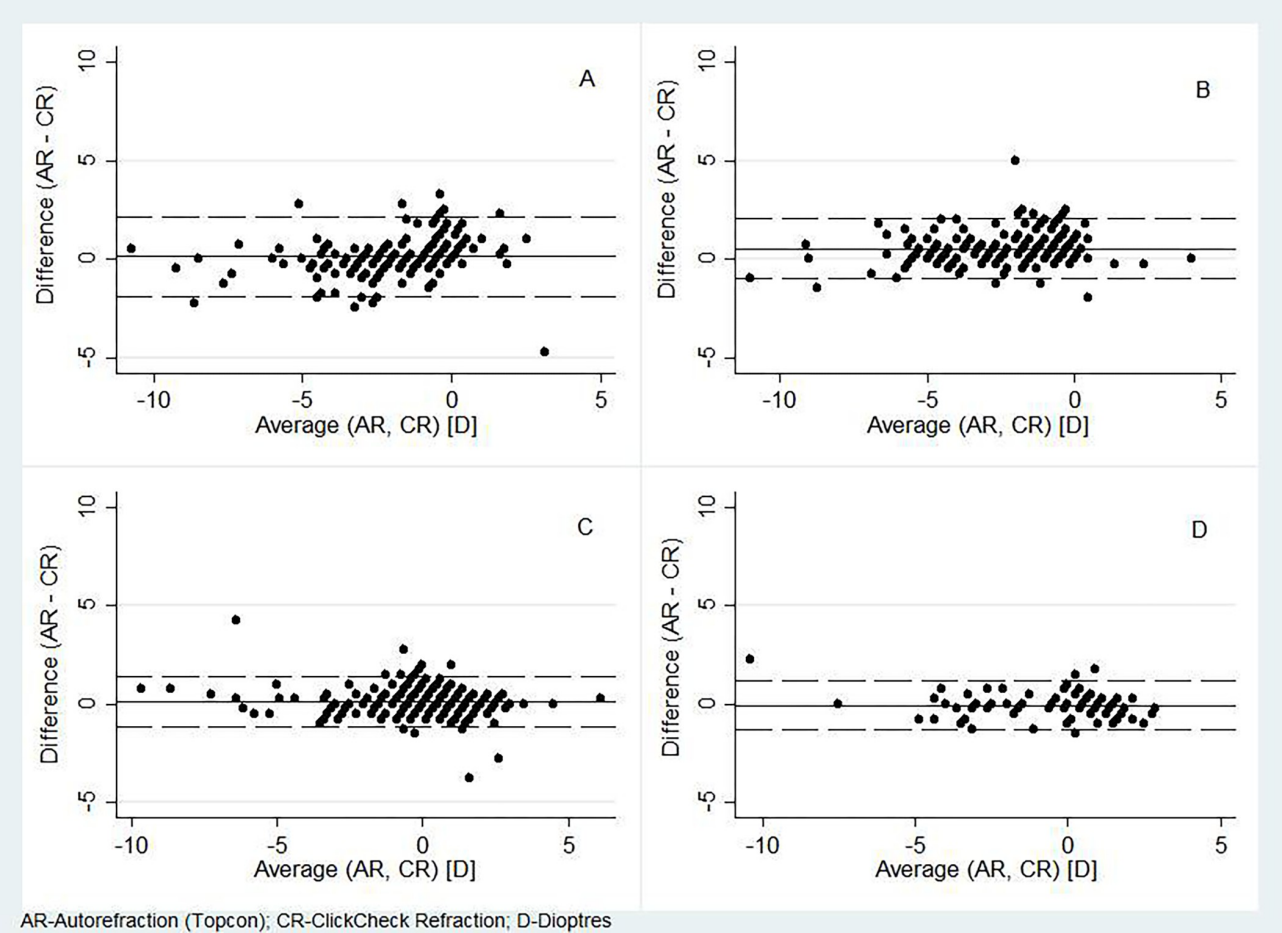
Bland Altman plot comparing the agreement of measurements by conventional autorefractor (AR) and ClickCheck^TM^ refraction (CR) across different age categories A) 7 to 15 years, B) 16 to 40 years, C) 41 to 59 years, D) 60 to 70 years.

In the subgroup analysis, the mean spherical refractive error was found to increase from the 7–15 age groups to 16–40 age group, then decrease considerably towards the 41–59 age group and then slightly increase in the 60–70 age groups [S1 Fig in S1 File]. This tread was found to be similar across the three refraction approaches except for a small increase between the first and second age categories in the case of AR [Table 2]. The ICCs >0.90 for all the three approaches across all categories denote a high level of agreement for measuring spherical refractive error amongst the three approaches [Figs 5 and 6]. These results are in line with those in the earlier analysis using pooled data. Comparison of visual acuity across the different age categories also showed moderate levels of agreement between SR and CR [Table 3].

**Table 2:** Comparison of spherical correction estimation using SR, AR and CR across different age categories.

**Spherical correction**	**SR**	**AR**	**CR**	**P value**	Comparison between all the three refractions	**ICC (95% CI)**	**Mean difference (95% CI)**	**LoA**
***7 to 15 years (n = 172)***	-1.45 (2.09)	-1.80 (2.42)	-1.92 (2.18)	0.126†		0.919 (0.891–0.940)^1^	0.472 (0.311 to 0.634)^1^	-1.679 to 2.623^1^
Mean (SD)								
Min—Max	-10.0 to 5.5	-10.5 to 3.0	-11.0 to 5.5			0.947 (0.929–0.961)^2^	0.119 (-0.035 to 0.273)^2^	-1.923 to 2.161^2^
***16 to 40 years (n = 249)***	-1.51 (1.99)^a^	-1.64 (2.14)^a^	-2.16 (2.01)	0.001†		0.935 (0.916–0.949)^1^	0.650 (0.554 to 0.745)^1^	-0.882 to 2.181^1^
Mean (SD)								
Min—Max	-10.0 to 2.0	-11.5 to 4.0	-10.5 to 4			0.949 (0.934–0.960)^2^	0.519 (0.424 to 0.615)^2^	-1.011 to 2.049^2^
***41 to 59 years (n = 438)***	0.06 (1.25)	0.26 (1.56)	0.11 (1.67)	0.117†		0.912 (0.893–0.927)^1^	-0.043 (-0.122 to 0.036)^1^	-1.722 to 1.637^1^
Mean (SD)								
Min—Max	-7.0 to 4.0	-9.25 to 6.25	-10.0 to 6.0			0.957 (0.948–0.964)^2^	0.157 (0.097 to 0.217)^2^	-1.124 to 1.438^2^
***60 to 70 years (n = 99)***	-0.36 (1.83)	-0.33 (2.24)	-0.27 (2.33)	0.951†		0.945 (0.918–0.963)^1^	-0.096 (-0.287 to 0.095)^1^	-2.008 to 1.816^1^
Mean (SD)								
Min—Max	-5.5 to 2.75	-9.25 to 2.75	-11.5 to 3.0			0.981 (0.972–0.987)^2^	-0.061 (-0.184 to 0.063)^2^	-1.300 to 1.179^2^

^1^Comparison between SR and CR

^2^Comparison between AR and CR, SR-Subjective Refraction; AR-Autorefraction (Topcon); CR-ClickCheck Refraction; ICC–Intra-class correlation, LoA–Limit of agreement, CI–Confidence Interval

†ANOVA

^a^No statistical significance was found between SR and AR in 16 to 40 years using Bonferroni corrections

**Table 3:** VA between SR and CR for various age categories.

Age category	Visual acuity		ICC (95% CI)	Mean difference (95% CI)	LoA
	SR	CR			
***7 to 15 years (n = 172)***					
Mean (SD)	0.08 (0.17)	0.21 (0.23)	0.676 (0.562–0.759)	-0.123 (-0.148 to—0.098)	-0.455 to 0.209
Min—Max	0–1	0–1			
***16 to 40 years (n = 249)***					
Mean (SD)	0.02 (0.09)	0.11 (0.16)			-0.377 to 0.190
Min–Max	0–1	0–1.18	0.412 (0.246–0.542)	-0.094 (-0.111 to -0.076)	
***41 to 59 years (n = 438)***					
Mean (SD)	0.05 (0.14)	0.18 (0.21)			-0.466 to 0.214
Min–Max	0–1	0–1.08	0.537 (0.44–0.616)	-0.126 (-0.142 to -0.110)	
***60 to 70 years (n = 99)***					
Mean (SD)	0.13 (0.17)	0.33 (0.25)			-0.597 to 0.200
Min–Max	0–0.6	0–1.18	0.451 (0.183–0.631)	-0.198 (-0.238 to -0.159)	

SR-Subjective Refraction; CR-ClickCheck Refraction; ICC-Intra-class correlation, LoA-Limit of agreement, CI-Confidence Interval, VA- Visual acuity

## Discussion

Refractive error is a common problem that has not been adequately addressed globally, resulting in an estimated $269 billion of lost productivity annually [1, 5, 18–20]. The current dearth of eye care professionals and high cost of conventional autorefractors warrant adoption of new strategies for refraction and spectacle prescription. This study demonstrates the feasibility of alternative methods for correction of refractive errors. Our results indicate a high level of accuracy for spherical refractive error measurements using CR with reference to gold standard subjective refraction among participants aged 7 to 70 years. The visual acuity achieved by the participants using corrections based on CR also was found to have moderate level of agreement with SR. This lends support to the use of the ClickCheck^TM^ device for first level screening before performing complete subjective refraction.

Epidemiological studies from various populations have established that the most prominent types of refractive errors are myopia followed by hyperopia [21–23]. Refractive errors may remain uncorrected primarily due to the lack of access to quality refractive services [24]. In this context, *ClickCheck*^TM^, an inexpensive and portable refraction device that can measure spherical refractive error accurately may contribute towards the diagnosis and treatment of distance visual impairment, especially in low- and middle-income countries.

The findings from the study suggest that the measurement of cylindrical power and axis with CR could be improved. The possible reasons for lower agreement in measurement of these parameters, apart from technological issues, may include difficulty differentiating between the blurred and sharp lines and the signs corresponding to axis on the vision chart used in the device. Even though astigmatism is less prevalent among children and adults compared to myopia or hyperopia [25], accuracy of astigmatism measurement by autorefractors has been evaluated and reported from various settings [26]. One important strength of this device is that the assessment of refraction is completely based on when a patient sees the letter ‘E’ clearly and not influenced by the prompting of a technician as in conventional practice. Further refinement of the technology and further research to establish accuracy may make the device more suitable for comprehensive measurement of refractive error. The moderate level of agreement for BCVA with vision corrected using CR and SR could be a reflection of the relatively small number of participants with significant astigmatism in the study sample.

Strengths of our study include a large and adequately powered sample size and the conduct of this study at a high-volume eye clinic in India. Masking of participants and those involved in data collection reduced the likelihood of bias in our findings. A primary limitation is the low rate of detection of astigmatism by *ClickCheck*^TM^. This restricted the validation of cylindrical power measurement by CR against SR and also restricted the comparison of spherical equivalent values of CR with the other two approaches (SR and AR). This indicates probable low sensitivity of ClickCheck to detect cylindrical correction and needs to be analysed in further studies. The device has a refractive range (spherical correction -11D to +8D and cylinder correction up to 4D) that covers a large majority of the population. Therefore, there is a possibility of high refractive errors being left out. However, the range of refractive corrections for majority of the participants included in our study was well within the measurable range of the device and those with high refractive errors may benefit from referral to an eye care provider since they are at higher risk for conditions like retinal tears and detachments or angle closure. It is encouraging to note that manufacturers from various settings have introduced autorefractors that utilises innovative technological approaches in order to improve refraction services. Many of them have also been reported to have good level of diagnostic accuracy, some of which are better than *ClickCheck*^TM^ [12, 27–29].

In conclusion, the assessment of spherical refractive error using the *ClickCheck*^TM^ had high agreement to SR and use of a conventional AR. The advantages of *ClickCheck*^TM^ over conventional autorefractors are its very low cost, portability, and ease of use with very minimal training. This approach may be suitable for enhancing refractive services in low resource settings. With refinement in the cylindrical power measurement, we believe that this device can play an important role in providing a first level assessment of refractive errors, prior to performing subjective refraction by trained personnel.

## Supporting information

S1 File (PDF)
