# Supplementary material for: Accuracy of a low-cost, portable, refractive error estimation device: Results of a diagnostic accuracy trial
Source: PLoS One. 2022 Aug 3;17(8):e0272451. doi: 10.1371/journal.pone.0272451 (PMC9348729; doi:10.1371/journal.pone.0272451)

Supplementary Table S1: Demographic details

Variables	
Age, mean (SD)	39.02 (17.94)
Age category, n (%)<br>7 – 15<br>16 – 40<br>41 – 59<br>60 – 70	 193 (17.9)<br>258 (23.9)<br>501 (46.4)<br>127 (11.8)
Gender, n(%)<br>Male<br>Female	 479 (44.4)<br>600 (55.6)
Refractive error >± 0.5D*<br>Yes<br>No	 477 (45.3)<br>576 (54.7)

Supplemental figure S1: Line graph showing the mean spherical correction by subjective refraction (SR), Topcon (AR) and ClickCheck^{TM} (CR) across different age categories

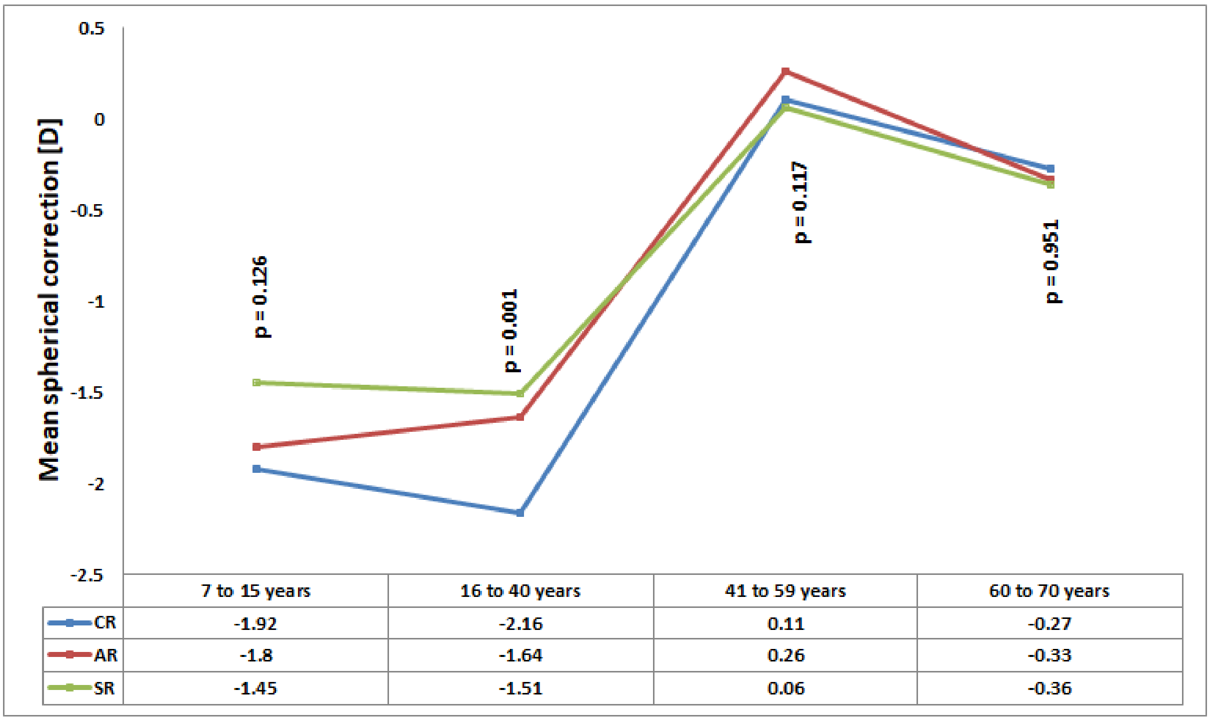

Supplement: S1 File — (PDF) [file pone.0272451.s001.pdf]
